# Evidence for the Vascular Origin of Benign Enhancing Foramen Magnum Lesions via Intraoperative Photographs: Case Report and Review of the Literature

**DOI:** 10.7759/cureus.20753

**Published:** 2021-12-27

**Authors:** Johannes Rosskopf, Bernd Schmitz, Meinrad Beer, Andrej Pala, Soung Yung Kim

**Affiliations:** 1 Neuroradiology, Bezirkskrankenhaus Guenzburg, Guenzburg, DEU; 2 Radiology, Ulm University, Ulm, DEU; 3 Neurosurgery, Bezirkskrankenhaus Guenzburg, Guenzburg, DEU

**Keywords:** vascular structure, intraoperative photographs, high signal lesion, foramen magnum lesion, benign enhancing lesion

## Abstract

A small, benign enhancing lesion posterior to the intracranial vertebral artery at the foramen magnum is a recently described image-based entity and believed to represent varix or ganglion. We report on an individual who underwent surgery due to a hybrid neurofibroma/schwannoma of the trigeminal nerve and additionally presented a small gadolinium-enhancing lesion in the right spinal canal at the level of the craniocervical junction (CCJ). The intraoperative finding of this enhancing lesion most likely represents the lateral internal vertebral venous plexus which does not require follow up or surgical excision.

## Introduction

A small, benign enhancing lesion posterior to the intracranial vertebral artery at the foramen magnum is an image-based newly described entity termed "benign enhancing foramen magnum lesion (BEFML)" [1]. It is assumed that this lesion might represent a venous finding, such as a varix, or a small nerve root ganglion/pseudoganglion. Due to the high risk of an operation and the benign appearance on imaging, there is no histological confirmation of BEFML so far.

This brief report presents an individual with a hybrid neurofibroma/schwannoma of the trigeminal nerve as well as a BEFML. To verify the above-mentioned hypotheses whether BEFML originates from varix or ganglion findings of the intraoperative situs were documented by intraoperative photographs and embedded by reviewing the literature.

## Case presentation

A 77-year-old female presented with a balance disorder and tendency to fall. Since December 2020, she reported intermittent dysphagia for solids and progressive dizziness. Except for daily ingestion of ACE inhibitors due to mild arterial hypertension there was no other previous medical history.

**Figure 1:**
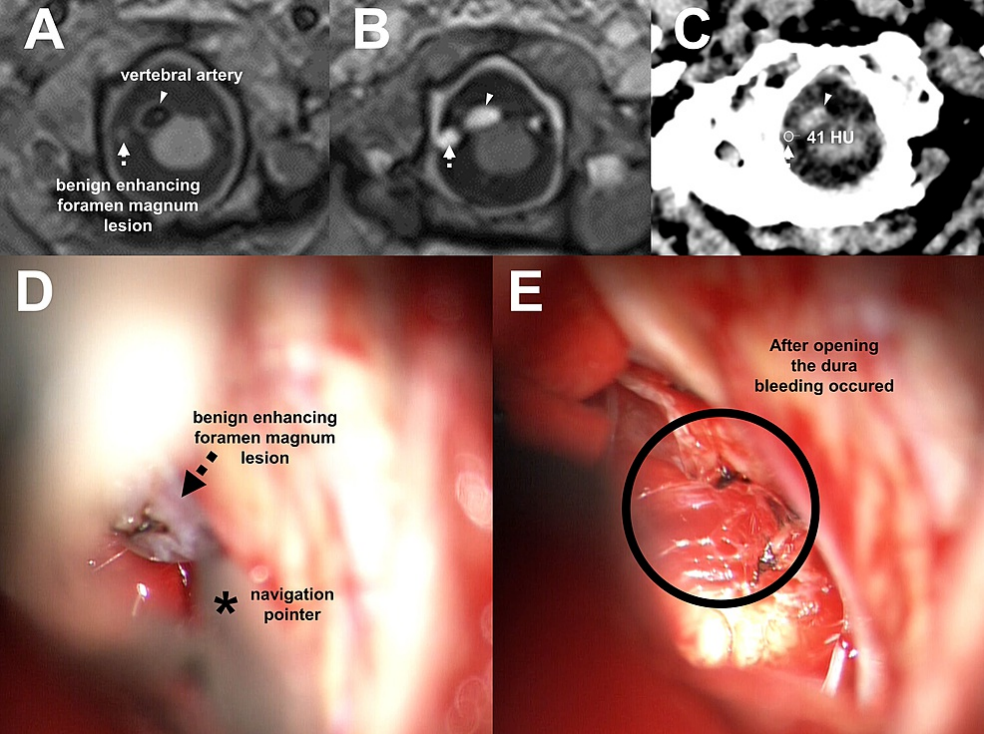
Case report of a benign enhancing foramen magnum lesion with intraoperative photographs On axial T1 volumetric non-enhanced (A) and postcontrast imaging (B) benign enhancing foramen magnum lesion (arrow) adjacent to the vertebral artery (arrowhead). The Hounsfield unit of this structure was 41 on non-enhanced brain CT (C). Images of surgical microscope (D and E). By use of a navigation pointer (D, asterisk) the benign enhancing foramen magnum lesion was identified as a nodular structure (D, arrow). The venous appearing structure was observed and the dura mater was opened. Neither a benign mass like a ganglion or a malign mass like a metastasis was found. Venous bleeding instead occurred (E).

In January 2021, computed tomography (CT) and magnetic resonance imaging (MRI) of the brain demonstrated a mass of the trigeminal nerve at the skull base which was assumed to cause the patient’s symptoms of dysphagia and dizziness. As an incidental finding, CT and MRI images showed additionally one sub-5 mm enhancing structure at the level of CCJ (Figures 1A-1C). This enhancing structure was independent of the cerebellopontine angle mass and remained image-morphologically unchanged in appearance in several follow-up scans resulting in the diagnosis of BEFML.

In March 2021, the patient underwent retrosigmoid craniotomy for resection of the cerebellopontine angle tumor. During the surgery, BEFML was also identified by the use of a navigation pointer and bleeding occurred after opening the dura there. Intraoperative photographic images were taken to capture the surgical view on BEFML (Figure 1, D and E).

The cerebellopontine angle tumor was histopathological classified as hybrid neurofibroma/schwannoma of trigeminal nerve. No histopathological analysis of BEFML tissue could be performed because neither a benign mass like a ganglion or a malign mass like a metastasis was found.

On the first day after surgery, the patient vomited and showed reduced vigilance. A control CT scan showed BEFML unchanged in appearance but bleeding was evident in the space of the resected cerebellopontine angle tumor with displacement of the brain stem accompanied by an increase in the volume of the cerebral ventricles. Consequently, there was an emergency surgery to relieve the bleeding and to install an external ventricular drainage system. Over the following days, the hydrocephalus and the compression of the brain stem decreased. However, as the patient presented on day five after surgery in clinical and laboratory-chemical examinations with the characteristics of a septic shunt infection intravenous antibiotics were administered for two weeks. Afterwards, the external ventricular drainage was changed to a ventriculoperitoneal shunting system and fortunately, the patient could be admitted in good general condition to a neurological rehabilitation clinic.

## Discussion

Accurate imaging diagnosis and evaluation of the foramen magnum/CCJ lesion is important since masses in this region could potentially impact adjacent critical structures such as the brain stem and upper cervical cord. True pathological intradural extramedullary lesions in the region of the foramen magnum/CCJ include meningioma, schwannoma, vertebral artery aneurysm, or malignancy/inflammatory disease of meninges. Cystic lesions, such as arachnoid cysts, neuroenteric cysts, and synovial cysts, have been also reported in the foramen magnum/CCJ [2]. A recent case series emphasized that benign lesions can also be presented in the foramen magnum / CCJ and were thought to represent a varix or ganglion [1].

Antonucci et al. [3] reported a case of the appearance of these lesions on magnetic resonance imaging (MRI) with intravenous contrast and labeled them as maybe venous in etiology. We recently observed the same finding in a 77-year-old woman undergoing a preoperative brain MRI for delineation of tumor extension. She presented additionally with a small gadolinium-enhancing lesion at the level of CCJ. Its image-based morphology was identical to previous reports of BEFML [1,2] supporting the benign nature of the process. The structure was visible and documented during the surgical approach to the cerebellopontine angle (Figure 1).

The microscopic view of the BEFML (Figure 1) indicated a venous plexus adjacent to the vertebral artery piercing through the dura. After opening the dura venous bleeding occurred and contact of the structure to the adjacent epidural venous plexus was visible. Due to bleeding, this contact could unfortunately not be documented adequately by photographs. However, this plexus ran parallel to the adjacent vertebral artery and was accessible caudally approximately to the tip of the dens, and cranially to the pyramis medullae oblongatae. This plexus is assumed to be connected with the lateral internal vertebral venous plexus [4].

This microscopic intraoperative finding is exactly in line with the previous image-based described entity termed BEFML. Table 1 summarizes the characteristics of other cases. In this previous case series and our case, a connection was recognized between the BEFML and an intradural vein connecting the internal vertebral venous plexus near the dural penetration of the vertebral artery [1,2].

This BEFML is adjacent to the expected course of a recently described venous structure termed "lateral internal vertebral venous plexus" [4]. This plexus is located in the lateral epidural space between the lateral masses of C1 and the base of the skull and was found in 33% of 15 adult cadaveric heads. This may be related to the lack of visibility of this BEFML in all patients.

**Table 1:** Reviewed article involving benign enhancing foramen magnum lesion BEFML: benign enhancing foramen magnum lesion

authors	Article type	main message	follow up	intra-operative proof	highlights
McGuinness et al. [1]	clinical report of 14 patients	first description of a newly recognized entity	in 9 patients	none	two patients underwent cerebral angiography and no arterial or venous structure were identified to explain the enhancing lesion on MR imaging
Antonucci and Spampinato [3]	letter to the editor	confirmed the presence of BEFML	18 months	none	BEFML might be associated with small bridging veins approximating nerve roots
Kogue et al. [5]	retrospective original article on 3717 patients	prevalence of high-signal lesions 3.4%	yes but not specified	none	possible association between high-signal lesion and spinal accessory nerve
Kogue et al. [6]	retrospective original article on 76 patients	3D balanced fast-field echo imaging distinguished between spinal accessory nerve and high-signal lesion	no	none	-
Nayate [7]	two case reports	enhancing lesion likely represents bridging veins	63 months and 1 year	none	-
Rosskopf et al. [present case]	case report	benign enhancing foramen magnum lesion is a venous structure documented on intraoperative photographs	no	yes	venous bleeding occurred during surgical investigation of the lesion

## Conclusions

BEFML is an image-based newly described entity. Due to the lack of histological confirmation, it was speculated whether it represents a varix or ganglion.

In our case, an individual presented with a hybrid neurofibroma/schwannoma of the trigeminal nerve as well as a BEFML. The microscopic intraoperative finding of our case raised evidence that BEFML is a venous structure most likely representing the lateral internal vertebral venous plexus. Clinicians should be familiar with BEFML to avoid unnecessary follow-up or potential surgical mishaps, even when assessing intracranial mass or metastatic disease.
